# Impaired Adipose Tissue Expandability and Lipogenic Capacities as Ones of the Main Causes of Metabolic Disorders

**DOI:** 10.1155/2015/970375

**Published:** 2015-04-02

**Authors:** Isabel Moreno-Indias, Francisco José Tinahones

**Affiliations:** ^1^Unidad de Gestión Clínica de Endocrinología y Nutrición, Instituto de Investigación Biomédica de Málaga (IBIMA), Complejo Hospitalario de Málaga (Virgen de la Victoria), Universidad de Málaga, 29010 Málaga, Spain; ^2^Ciber Fisiopatología de la Obesidad y Nutrición (CIBEROBN), 28029 Madrid, Spain

## Abstract

Obesity is considered a major health problem. However, mechanisms involved and its comorbidities are not elucidated. Recent theories concerning the causes of obesity have focused on a limit to the functional capacity of adipose tissue, comparing it with other vital organs. This assumption has been the central point of interest in our laboratory. We proposed that the failure of adipose tissue is initiated by the difficulty of this tissue to increase its cellularity due to excess in fat contribution, owing to genetic or environmental factors. Nevertheless, why the adipose tissue reduces its capacity to make new adipocytes via mesenchymal cells of the stroma has not yet been elucidated. Thus, we suggest that this tissue ceases fulfilling its main function, the storage of excess fat, thereby affecting some of the key factors involved in lipogenesis, some of which are reviewed in this paper (PPAR*γ*, ROR1, FASN, SCD1, Rab18, BrCa1, ZAG, and FABP4). On the other hand, mechanisms involved in adipose tissue expandability are also impaired, predominating hypertrophy via an increase in apoptosis and a decrease in adipogenesis and angiogenesis. However, adipose tissue failure is only part of this great orchestra, only a chapter of this nightmare.

## 1. Introduction

Obesity is considered a major health problem and its prevalence has increased dramatically in the last decades, corresponding to the 36.9% of the men and the 38.0% of women worldwide [1]. Obesity is usually accompanied by other diseases, the most common being type 2 diabetes mellitus (T2D) [2], insulin resistance (IR) [3], and cardiovascular complications [4]. Moreover, other metabolic consequences have been related, such as nonalcoholic fatty liver disease (NAFLD) [5] or even many cancers [6]. Thus, obesity must be studied from a global perspective.

Although obesity is not the unique problem in metabolic syndrome, this is becoming more common due to a rise in obesity rates among adults [7]. Over the last few years, the number of people with diabetes mellitus has increased massively, raising prevalence worldwide, and does not focus on the western societies. In the economically advanced countries, the increase will be about 50% in 2030 [8], so it has become one of the most important public health challenges globally. Obesity is a major cause of T2D [9]. Although great advances have been made in understanding the mechanisms involved in the pathogenesis of T2D, these have still not been fully elucidated. Currently, the only effective therapy is weight loss, including the lately accepted bariatric surgery [10], and last knowledge about the effectiveness of the physical activity, which is able to decrease specific visceral fat increasing the fat-free mass [11].

Adipose tissue is a key regulator of energy balance, playing an active role in lipid storage and buffering, synthesizing, and secreting a wide range of endocrine products into circulating blood that influence systemic metabolism [12]. A classical paradigm has been the fact that the more adipose tissue, the higher the prevalence of metabolic diseases [13], and it is this relationship that has interested researchers. However, certain inconsistencies have been found; for example, some extremely obese people are not diabetic, while other overweight people develop severe IR and diabetes [14]. This suggests that the absolute amount of fat stored may not be the most important factor determining the relationship between obesity and diabetes. Obesity-related adverse health consequences therefore appear to be related to fat distribution rather than the total amount of fat [15].

There are many theories linked to obesity: from the simplest excess of energy/lipids intake to the most recent gut microbiota [15, 16]. But a very interesting theory is focused on a limit to the functional capacity of adipose tissue. When this capacity is exceeded, metabolic disorders occur [17]. This suggests that the factor linking obesity, diabetes, and associated comorbidities may not be the absolute amount of fat accumulated but the mismatch between energy surplus and storage capacity [18]. This new vision places the adipose tissue at the same level as other vital organs. Just as one can speak of heart, liver, or kidney failure, we propose the idea of adipose tissue failure.

The aim of this review is to better understand the adipose tissue organ, reviewing the influence of variables that govern its ability to expand and its influence on development of IR and diabetes associated with obesity, mainly based on the studies undertaken by our research team over the last few years.

## 2. Lipogenic Capacity of Adipose Tissue

Fat accumulation is determined by the balance between fat synthesis (lipogenesis) and fat breakdown (lipolysis/fatty acid oxidation). Lipogenesis encompasses the processes of fatty acid synthesis and triglyceride synthesis, and takes place in both the liver and in adipose tissue. It has long been accepted that the primary function of adipocytes is to store fuel for distribution to nonadipose tissues in times of need [19], but Adipose tissue is also an important site of endogenous fatty acid synthesis, although lipogenesis in this tissue is considered low and less than that in the liver [20].

Excessive hepatic lipogenesis is a hallmark feature of many models of obesity and diabetes, although the causal relationship between tissue lipid accumulation and insulin resistance is unclear [21]. Some of the most feasible reasons are its relationship with NAFLD [5], endoplasmic reticulum (ER) stress [22], the role of the free fatty acids (FFA) [23] or/and ceramides [24].

Lipogenesis is thought to be a relatively minor contributor to whole body lipid stores in a present-day human consuming a typical high fat diet [25]. Nevertheless, under special situations such as a high carbohydrate diet, the total body fat synthesis significantly exceeds de novo lipogenesis [26]. It is well recognized that adipose tissue storage capacity for fatty acids prevents lipotoxicity in other tissues [27]. However, adipose tissue buffering is impaired in obesity through defects in the ability of adipose tissue to respond rapidly. Thus, the lipogenic pathway has been suggested to be downregulated in obesity, at least at the gene expression level [28]. Although in early obesity there is an increase in lipogenic gene expression to store fat rapidly, in long-term obese subjects this expression decreases, perhaps due to a late adaptive process, aimed at limiting further development of fat mass [29]. A possible explanation for this reversal is that once the storage capacity of the adipocytes is reached, the cells reduce their ability to synthesize additional fatty acids, following a natural inhibitory feed-back process [28].

### 2.1. Key Factors Implicated in Adipose Tissue Lipogenesis

**Figure 1:**
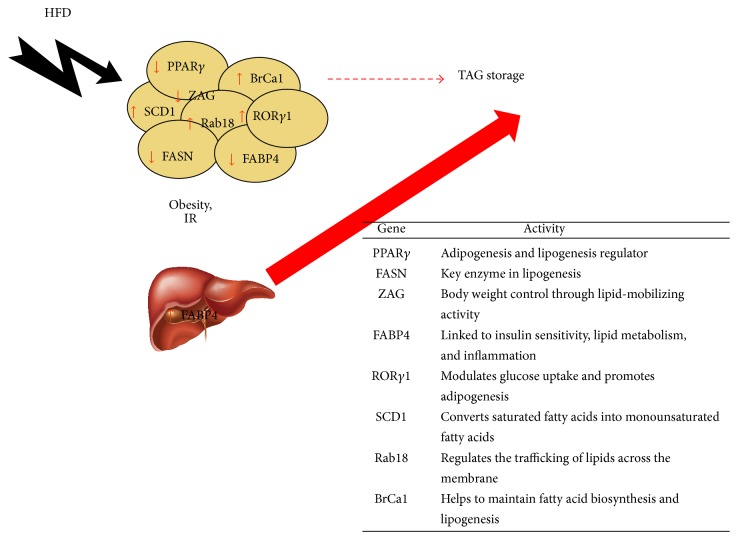
Lipogenic pathway in obese patients. The loss of the lipogenic capacity of the adipose tissue is compensated by other tissues and organs, mainly the liver. Thus, many molecules come into play in this loss of lipogenic capacity of the adipose tissue, acting in a wrong way. HFD: high fat diet; IR: insulin resistance; TAG: triacylglycerol; PPAR*γ*: peroxisome proliferator-activated receptor-*γ*; FASN: fatty acid synthase; ZAG: zinc-*α*2 glycoprotein; FABP4: fatty acid-binding protein 4; ROR*γ*1: retinoic acid receptor-related orphan nuclear receptor *γ*1; SCD1: stearoyl-CoA desaturase-1;* Rab18*:* Ras-related protein 18*; BrCa1: breast cancer 1.

Thus, changes in lipid-storing capacity and lipid mobilization processes in adipocytes represent important elements of adipose tissue dysfunction. Many factors are involved in this action, although we will focus on those our research team has been working on (Figure 1) in the last years, some of them are widely known, others are shown as a novel approach. Gene expression patterns play a key role in determining pathogenesis and candidate genes of T2DM and obesity [30, 31], because of their first step in the transcription and function cascade. Thus, many of the presented results are base on this technique.

#### 2.1.1. Peroxisome Proliferator-Activated Receptor-*γ* (PPAR*γ*)

Peroxisome proliferator-activated receptor-*γ* (PPAR*γ*) is a regulator of adipogenesis and lipogenesis, being considered the most important regulator of these processes. The behavior of PPAR*γ* has long been recognized from clinical, pathological, observational and case studies. The activation of PPAR*γ* leads to adipocyte differentiation and fatty acid storage, whilst it represses genes that induce lipolysis and the release of free fatty acids (FFA) in adipocytes [32]. Failure in the metabolism of this molecule leads to dysregulation in the optimal lipid storage and mobilization, the main problem of obesity. Under normal conditions, PPAR*γ* mRNA expression is highest postprandially and its activation leads to upregulation of genes that mediate fatty acid uptake and trapping, ensuring the storage and relocalization of the excess triacylglycerol [33]. Moreover, PPAR*γ* has a direct role in the transcriptional control of specific functional nodes of the lipolytic axis through the protein kinase A (PKA) complex [34]. On the other hand subjects with IR and obesity have a reduced PPAR*γ* expression, both fasting and postpandrially [35, 36]. Morbidly obese patients and patients with diabetes have a lower expression of PPAR*γ*2 mRNA in comparison with morbidly obese insulin sensitive patients, both in VAT and muscle [36]. This is closely associated with the storage capacity in adipose and muscle tissues, with the mismatch between energy surplus and storage capacity in adipose tissue and muscle tissues possibly being an important factor linking obesity and IR [37]. This lower expression was also found in both PPAR*γ* and PPAR*γ*2 mRNA in peripheral blood mononuclear cells after a high-fat meal in morbidly obese humans and it was more patent in those who were insulin resistant, indicating an altered response in these individuals [35].

#### 2.1.2. Fatty Acid Synthase (FASN)

On the other hand, another important molecule in this process is the fatty acid synthase (FASN), which is the central enzyme for de novo synthesis of long-chain saturated fatty acids, and is a key enzyme in lipogenesis. FASN expression and activity are regulated by insulin [38]. So from this point, concerning carbohydrate metabolism disorders and obesity, we have demonstrated that adipose FASN gene expression is closely related with the hyperglycemic state (higher in normoglycemic individuals compared with those with hyperglycemia) [39]. In addition, we have also demonstrated a significant decrease in FASN expression in hypertensive individuals, being more pronounced in obese patients [40].

#### 2.1.3. Zinc-*α*2 Glycoprotein (ZAG)

Another protein recently studied is Zinc-*α*2 glycoprotein (ZAG), a protein expressed in mature adipocytes and in VAT and SAT and which is involved in body weight control through its lipid-mobilizing activity via interaction with *β*3-adrenoreceptors, suggesting a role in lipid catabolism [41]. ZAG may therefore be considered an adipokine. Previous in vivo studies showed that ZAG induces a loss of body weight in overweight and obese animals through specific depletion of carcass fat [42]. Studying the most extreme form of obesity, we found an inverse relationship between ZAG expression and body mass index. We suggested that ZAG may be involved in the regulation of lipid metabolism due to a different expression in VAT and SAT and its different relation with the genetic expression of lipolytic enzymes [43].

#### 2.1.4. Fatty Acid-Binding Protein 4 (FABP4)

Another way to address the issue of the loss of lipogenic capacity in individuals with IR is the compensation of that loss by tissues and organs other than the adipose tissue. For instance, fatty acid-binding protein 4 (FABP4), a protein linked to insulin sensitivity, lipid metabolism and inflammation [44], has been described as an important mediator in the crosstalk between adipocytes and macrophages in adipose tissue. We have suggested that the adipose tissue and the liver may act in a balanced manner depending on the IR status, as a decrease in FABP4 expression was observed in adipose tissue from metabolically obese patients versus metabolically healthy humans, while the opposite takes place in the liver, with an increase in FABP4 expression in metabolically obese patients, with the IR status being an important determinant in tissue expression [45].

#### 2.1.5. Retinoic Acid Receptor-Related Orphan Nuclear Receptor *γ*1 (ROR*γ*1)

Another orphan nuclear receptor associated with IR, and which has been studied in our laboratory, is the retinoic acid receptor-related orphan nuclear receptor *γ*1 (ROR*γ*1), also expressed in human adipose tissue. ROR*γ*1 has an important role in adipogenesis and glucose homeostasis, modulating glucose uptake and promoting adipogenesis [46]. Ror*γ*(−/−) mice are protected from hyperglycemia and IR in the state of obesity [47]. Subjects with obesity and a high degree of IR have a clear rise in gene and protein expression of the ROR*γ*1 receptor in adipose tissue, especially in the visceral depot. This suggests that ROR*γ*1 may be added to the list of nuclear receptors in adipose tissue that could be used to modulate the IR associated with obesity [48].

#### 2.1.6. Stearoyl-CoA Desaturase-1 (SCD1)

Stearoyl-CoA desaturase-1 (SCD1), which is an endoplasmic reticulum-bound enzyme that converts different saturated fatty acids into mono-unsaturated fatty acids [49]. Studying the regulation of SCD1 is of particular interest since alterations in the composition of phospholipids have been implicated in a variety of diseases, including cancers, diabetes and cardiovascular disorders [50]. Mice with a targeted disruption in the scd1 gene are resistant to diet-induced weight gain [51]. In accord with these results, we have reported that SCD1 in morbidly obese patients is related to obesity and IR, with a raised SCD1 protein level in VAT and SAT from morbidly obese patients [52]. A recent collaboration has found an adaptive response to compensate the antilipogenic effect associated with IR, maintaining lipid desaturation through preferential SCD1 regulation and facilitating fat storage in adipose tissue [53].

#### 2.1.7. Rab18 (Ras-Related Protein 18)

Another example of up-regulated molecules in obesity is Rab18, which is a GTPase that has been found to regulate intracellular membrane bidirectional trafficking of lipids in lipid droplets [54]; it is involved in the mechanism to release lipids from lipid droplets in adipocytes. Rab18 overexpression increased basal lipogenesis and Rab18 silencing impaired the lipogenic response to insulin, suggesting that this protein promotes fat accumulation in adipocytes, performing its activity through the endoplasmic reticulum [55]. On the other hand, there is evidence for the participation of Rab18 in the regulation of lipolysis via *β*-adrenergic pathway [56]. Moreover, obesity is associated with an increase in Rab18 expression, which suggests that upregulation of this GTPase may be an appropriate response to managing energy excess, an adaptive response to overcome the alterations in lipid metabolism occurring in obesity [55]. This participation in the regulation of lipid processing in adipose tissue takes place under both normal and pathological conditions [57].

#### 2.1.8. Breast Cancer 1 (BrCa1)

And finally, we finished with Breast Cancer 1 (BrCa1), a good example of a protein with an important role in the lipogenic and lipolysis pathways involved in the relationship between obesity and its associated metabolic pathologies BrCa1 is a protein involved in multiple cellular functions, including DNA repair, cell cycle checkpoint control, and transcription associated with the DNA damage as it has been reported in breast and ovarian cancers [58], and ubiquitination, activity developed thanks to the heterodimer with BARD1 [59], among others. It has been suggested that BrCa1 helps to maintain fatty acid biosynthesis and lipogenesis under control [60]. This is supported by findings from our laboratory showing an increased BrCa1 expression in adipose tissue and during adipogenesis that mirror the effects of lipogenic enzymes such as acetyl-CoA carboxylase (ACC) and FASN [61]. On the other hand, BrCa1 was found to be up-regulated in adipose tissue from obese subjects independently of whether they had T2D, so we suggest a crosstalk between BrCa1, lipogenesis, adipogenesis, obesity, and obesity-associated IR. Thus, taking together the increased expression of the BrCa1 gene and the reduced expression of many lipogenic factors, could be interpreted as the process through which cells reduce their ability to synthesize additional fatty acids once the limit in the storage capacity of the adipocytes is attained.

## 3. Expandability of the Adipose Tissue

Adipose tissue, as suggested above, can be considered another vital organ, mainly due to its endocrine properties. Adipocytes are the main units in adipose tissue, and this tissue is considered to control the whole body metabolism, so dysregulation can have huge consequences for health. Adipose tissue dysfunction is thought to be the major factor leading to whole body IR [62]. Beyond expandability of adipose tissue, the essential pathological changes of adipose tissue affect whole body energy homeostasis and integrity.

### 3.1. Neogenic Capacity of the Adipose Tissue

**Figure 2:**
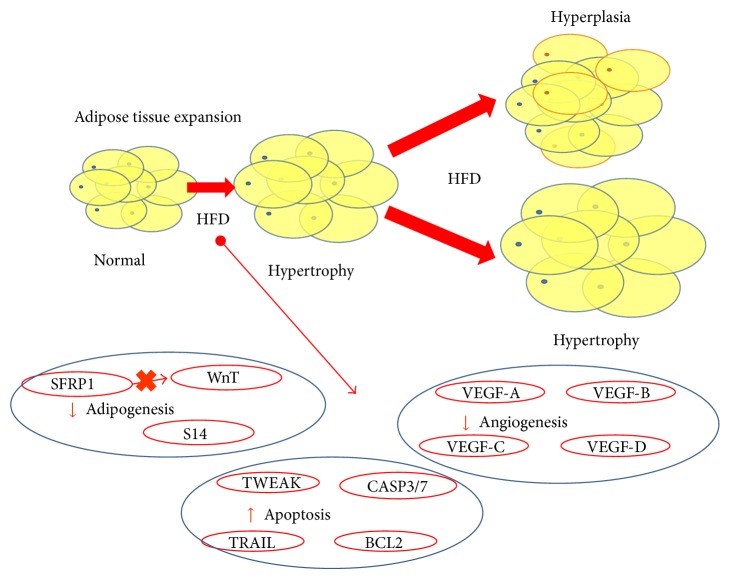
The adipose tissue expansion is produced via hypertrophy in a first point. If the surplus of energy continues, adipose tissue expansion will happen via hyperplasia and/or hypertrophy. In these mechanisms, different mechanisms intervene: adipogenesis, apoptosis, and/or angiogenesis, which will determine the metabolic status of the patient. Moreover, within each mechanism, this adipose tissue can be modulated. HFD: high fat diet; SFRP1: secreted frizzled-related protein 1; S14: thyroid hormone responsive Spot 14; VEGF-A/B/C/D: vascular endothelial growth factor A/B/C/D; TWEAK: TNF-like weak inducer of apoptosis; CASP3/7: Caspasas3/7; BCL2: B-cell lymphoma 2; TRAIL: TNF (tumor necrosis factor)-related apoptosis-inducing ligand.

Adipose tissue is very dynamic; as much as 10% of the fat cells die and are renewed every year [63]. Lipid excess in AT results in increased adipocyte size (hypertrophy) or/and increased numbers of adipocytes (hyperplasia), seen as enlarged SAT or VAT [64] (Figure 2).

As it has been noticed, there are metabolic and functional differences between adipose tissue depots; SAT is recognized as the safest TAG depot, and it is the first to receive excess lipids, whereas visceral deposition occurs only after SAT capacity has been exceeded [65]. Thus, VAT has a greater capacity to generate free fatty acids and to uptake glucose, while SAT is more avid in the absorption of circulating free fatty acids and triacylglycerols [66].

On the other hand, a different impact of subcutaneous and visceral fat cell sizes on lipid and glucose/insulin profiles has been observed [67]. So that, mean fat cell size is associated with metabolic complications but not with systemic or adipose inflammation in morbid obesity, with a region-specific influence: large visceral fat cells are more strongly linked to dyslipidaemia, whereas large subcutaneous fat cells correlate with impaired glucose metabolism, hyperinsulinaemia and insulin resistance [67]. Thus, Veilleux et al. [68] suggested that VAT, but not SAT, adipocyte hypertrophy is associated with an altered lipid profile independent of body composition and fat distribution in women. These differences may be determinant in the development of obesity and related disorders, with IR being the most important factor linking VAT to cardiovascular risk [66]; whereas in states of low IR the expression of most of the enzymes in the lipogenic and lipolytic pathways is greater in SAT facilitating triglyceride/fatty acid cycling [69].

However, the large interindividual variability observed in adipocyte size at a given adiposity level suggests that the tendency to fat cell hypertrophy in each fat compartment may differ among individuals [68].

#### 3.1.1. Molecular Approximation

**Figure 3:**
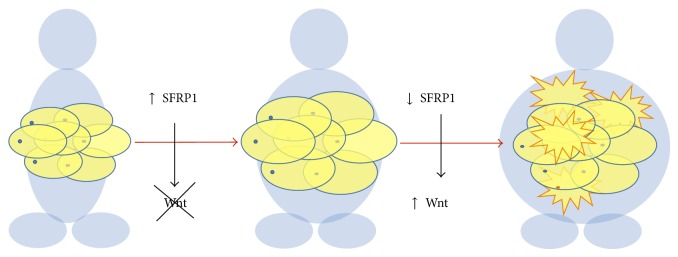
Secreted frizzled-related protein 1 (SFRP1) upregulation in early stages of obesity facilitates adipose tissue expansion, falling in morbidly obese patients [77].

Although it may seem incongruent, strategies that increase the capacity of adipose tissue to store lipid and, therefore, make individuals more obese may in fact confer metabolic benefits [70]. Allowing adipose tissue to store more lipids may prevent secondary metabolic complications caused by lipids being deposited in nonadipose organs [71]. Thus, the ability of adipose tissue to expand and match the storage needs of energy surplus may be a key determinant in protection against the metabolic syndrome associated with obesity [72]. It is therefore very important to understand the signaling factors that control adipose tissue expansion. The Wnt family is known to control adipocyte differentiation, and several members of the Wnt family have been shown to inhibit the early steps of adipogenesis. Conversely, endogenous inhibitors of Wnt signaling were found to promote the generation of adipocytes [73]. One group of these extracellular Wnt antagonists is secreted frizzled-related proteins (SFRPs, also known as secreted apoptosis-related proteins or SARPs) [74]; at least five structurally similar SFRPs have been identified. In obesity it has been observed that mRNA levels of SFRP1-4, but not SFRP5, were altered; finding that SFRP1, SFRP2 and SFRP4 are adipokines and their expressions correlated with insulin sensitivity [75]. However, it has been demonstrated that, in the setting of obesity, SFRP5 secretion by adipocytes exerts salutary effects on metabolic dysfunction by controlling inflammatory cells within adipose tissue [76]. On the other hand, there is limited evidence to support a role for endogenous SFRP1 in the physiological and/or pathological development of human obesity and the metabolic syndrome. Accordingly, in collaboration with our team Lagathu et al. [77] suggested a model of adipose tissue expansion characterized by upregulation of SFRP1 in the early stages of obesity. This elevation of SFRP1 could inhibit Wnt signaling and, therefore, facilitate adipose tissue expansion, allowing nutrient storage demands to be met. Further weight gain results in SFRP1 levels falling back to the same values as lean subjects, and limiting adipogenesis through increased Wnt/*β*-catenin signaling, compromising further adipose tissue expansion (Figure 3).

Nevertheless, new factors have been discovered that induce adipogenesis, such as thyroid hormone responsive Spot 14 (THRSP or S14), another specific factor whose gene expression and protein levels in lipogenic tissues are strongly linked to glucose thyroid hormone, insulin, and glucose and that is directly associated with adipogenesis in human adipocytes but inversely related to obesity and omental fat accumulation [78].

The great revolution in the study of the adipose tissue, though, has been the isolation and characterization of an adipose tissue-derived multipotent stem cell population (hO-MSC). This has opened up new possibilities, especially concerning the study of how this multipotent stem cell population resident in the adipose tissue is affected by, or contributes to, tissue dysfunction [79]. It is therefore important to evaluate the influence of the adipogenic environment. Several studies have focused on the impaired preadipocyte differentiation in obesity. Isakson et al. [80] observed that preadipocytes from obese individuals present a reduced adipogenic potential correlating with an increasing BMI, while Cleveland-Donovan et al. [81] observed alterations in the proliferative ability by impaired cell cycle activation. In accordance with these observations, we demonstrated an association between obesity and a loss of stemness in the hO-MSC population, featured by impaired multilineage differentiation potential in the stem cell regulatory network. hO-MSCs from obese subjects have greater senescence and this senescence is increased as the degree of obesity rises. Thus, obese subjects have a lower differentiation capacity of their hO-MSC to adipose and bone tissue [82]. Several molecular pathways may explain the refractory response of hO-MSCs of morbidly obese patients to differentiation. In summary, despite a potential genetic disposition to force the adipogenic cell fate by the upregulation of PPAR*γ*, the combination of dysregulated genes (mainly members of Notch and SHH, FOXA2 and FOXC2; inhibitors of adipogenic differentiation) and miRNAs (miRNA23b and miR27b, which are PPAR*γ* and CEBP*α* repressors and Wnt activators, and miR103, miRNA542-5p, and miRNA320, involved in Wnt dependent inhibition of adipogenesis, among others) may cause a block, inducing a failure to enter and/or progress to the adipogenic fate [82]. Thus, hO-MSCs from morbidly obese subjects have an impaired capacity to expand and differentiate to other features. This is reflected in the so-called “adipose tissue expandability hypothesis,” where the pathological expansion of abdominal adipose tissue in morbid obesity reaches a threshold characterized by an inability of adipose tissue to expand because its capacity to recruit new adipocytes is exhausted. This is associated with metabolic complications and IR due to ectopic deposition of excess lipid in nonadipose tissue [83].

#### 3.1.2. Apoptotic Capacity of the Adipose Tissue

Apoptosis is a fundamental mechanism for the homeostasis of mammalian tissues and it has been linked to a variety of disorders. Apoptosis is a form of programmed cell death that occurs under certain physiological and pathological conditions as a common mechanism of cell replacement, tissue remodeling, and elimination of damaged cells. The dysregulation of this process has been suggested to contribute to obesity, differences in regional fat distribution, or lipodystrophy [84]. Recently, a relationship between adipose tissue inflammation and apoptosis has been proposed [85, 86], although apoptosis of adipose tissue is still a relatively poorly studied phenomenon.

Many proapoptotic and antiapoptotic molecules are mediated in apoptosis, achieving homeostasis of the mammalian tissues. Modulation of apoptosis is emerging as a promising antiobesity strategy because removal of adipocytes through this process will result in reducing body fat [87]. Two of the main families involved in apoptosis are the caspases and B-cell lymphoma 2 (BCL2) proteins. Recently, we found an increase in proapoptotic CASP3/7 gene expression and a decrease in antiapoptotic BCL2 gene expression in adipose tissue (both VAT and SAT) with the increase in body fat mass [88]. Moreover, in vitro studies demonstrated that culture with proinflammatory factors from adipocytes increases the apoptotic pathway. These phenomena could be as a consequence of obesity-induced inflammation; thus we linked these results with a state of IR as these changes were paralleled by an increase in gene expression of inflammatory cytokines (TNF-*α* and IL-6) and macrophage infiltration markers [88].

Many markers have been associated with apoptosis, mainly through inflammation, some with proapoptotic and others with antiapoptotic properties. A multifunctional proapoptotic cytokine belonging to the TNF superfamily, named TNF-like weak inducer of apoptosis (TWEAK), controls many cellular activities and has emerged as a new player in the inflammatory process. TWEAK (and its receptor Fn14) is upregulated in severe obesity, because of the modulation of the microenvironment by the infiltrated macrophages [89] and not by hypoxia [90]. In a recent collaboration, we found that a decrease in the soluble form of TWEAK in severely obese patients may favor the proinflammatory activity of TNF*α* [91].

The latest studies have shown that TRAIL [TNF- (tumor necrosis factor-) related apoptosis-inducing ligand] ameliorates the natural history of diabetes mellitus, associating the changes induced by a significant reduction in proinflammatory cytokines with a modulation of adipose tissue gene expression and apoptosis [92]. Thus, circulating TRAIL levels may indicate the severity of T2D; low circulating levels may precede the onset of T2D whereas higher levels of soluble TRAIL may indicate chronic T2D [93]. Moreover, by binding to TRAIL-R2, TRAIL activates the cleavage of caspase-8 and caspase-3, which in turn cleaves and inactivates PPAR*γ*. This causes changes in gene expression of lipogenic genes, such as GLUT4, FASN, and ACC, and finally leads to the inhibition of insulin-stimulated glucose uptake and lipogenesis [94]. This reduction in PPAR*γ* also reduces adipocyte differentiation [94], consistent with the study of Bernardi et al. [92] who attributed the improvement of metabolic abnormalities of T2D following TRAIL treatment, with its effect on the adiposity, which might then have influenced proinflammatory cytokines levels and glucose metabolism.

#### 3.1.3. Angiogenesis of the Adipose Tissue

The development and maintenance of fat depots require angiogenesis. Adipose tissue is highly vascularized, and each adipocyte is nourished by an extensive capillary network. Thus, adipose tissue expansion is linked to the development of its vasculature [95]. However, in hypertrophy, the increase in vascularization does not happen in parallel. Normal angiogenesis depends on the intricate balance between angiogenic and angiostatic factors. Molecular mechanisms of switching in angiogenic phenotypes, in both healthy and pathological tissues, involve an imbalanced production of overlapping angiogenic factors and inhibitors [96]. It has been documented that abnormalities of angiogenesis may contribute to the pathogenesis of diabetes complications.

The vascular endothelial growth factor (VEGF) and its receptors play a crucial role in both angiogenesis and vasculogenesis. VEGF has also been recognized as being a potent stimulator of endothelial proliferation and migration [97]; moreover it has been noticed that its signaling is needed for an adequate adipose tissue function [98]. White adipose tissue produces and secretes many different types of proangiogenic factors, such as VEGF-A and VEGF-B: the two key angiogenic factors produced by adipocytes [99]. Other adipose-tissue derived factors with proangiogenic properties include VEGF-C and VEGF-D, which have been found to be important for the proper formation and maintenance of the lymphatic network. Overexpression of VEGF resulted in increased blood vessels number and size in white and brown adipose tissues [100]. In our laboratory, much interest has been generated after finding that morbidly obese subjects with low insulin resistance had higher adipose tissue VEGF-A levels than obese subjects with high insulin resistance, hypothesizing that upregulation of VEGF-A in adipose tissue could have a relationship with IR, believing that its upregulation is a compensatory mechanism that replaces the reduction in VEGF-B, VEGF-C, and VEGF-D [101]. Other complications derived from diabetes include dyslipidemia and atherosclerosis, and VEGF has also been related with these disorders, with VEGF-C rather than VEGF-A being more closely related [102].

## 4. Discussion

The data presented in this paper, however, is only a little portion of the problem. Old trends tended to focus on one part, but now we know that everything is much more complex. In obesity, there is not a single cause, due to obesity is a part of a major problem, metabolic syndrome and moreover, other comorbidities as diabetes or cardiovascular diseases. And, for that reason, we must try to understand the problem as a complicated network in which all the pathways are related.

In this work, we have focused on the pathologies associated with an adipose tissue failure from a molecular approach, but this is only a tiny part of the story. Thus, summing up, many are the genes that have been related to obesity and its comorbidities such as T2D. PPARg continues being the central actor of the scene, but the dysfunction of many others has been recognized as part/cause of the development of these diseases; some of them belong to the lipogenesis pathway (FASN, FABP4, and SCD1) and others used to be associated with other roles (ZAG, RORa1, Rab18, and BrCa1), but now their roles in this dysfunction are emerging. But lipogenesis is not the only process involved; other crucial processes for the normal function of the adipose tissue are adipogenesis, apoptosis, and angiogenesis: necessary processes to help in the adipose tissue expansion in order to avoid future metabolic disturbances caused by the excess of fatty acids. SFRP1 and Spot14 are two genes influenced by the weight gain, involved in adipogenesis, and whose overexpression protects from metabolic disturbances. However, it has been noticed that morbidly obese patients have this process endangered in the same way that occurs with caspases and BCL2 proteins, or TWEAK and TRAIL, concerning to apoptosis, or with the VEGF family in the case of angiogenesis, which are also deregulated in morbidly obese patients and to the increase of fat.

**Figure 4:**
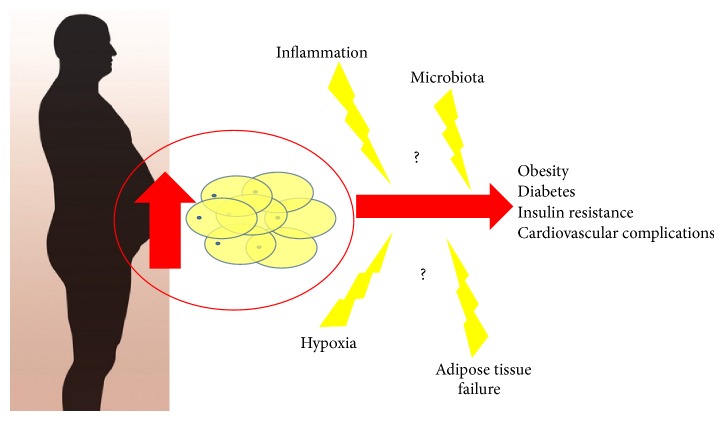
Fat accumulation leads to a high number of commorbidities associated to the metabolic syndrome. Many factors intervene in this situation. Many of them are known but not understood, many are even unknown, so research must be continue until we can elucidate how this great orchestra sounds in the right way.

Therefore, although obesity has been mainly related to perturbations of the balance between food intake and energy expenditure, other factors must nevertheless be considered (Figure 4). At present, many other hypotheses have been developed trying to explain the causes of the adipose tissue hypertrophy and hyperplasia. A hot topic, for example, is the relationship with the gut microbiota; nowadays there is a growing interest in elucidating how these microorganisms can help or aggravate the problem. Various mechanisms have been proposed to explain the influence of the microbiota on metabolic disorders, such as metabolic endotoxemia, modifications in the secretion of the incretins, and butyrate production [16]. Metabolic endotoxemia is generated by the lipopolysaccharides (LPS): endotoxins commonly found in the outer membrane of Gram-negative bacteria [103]. These LPS are absorbed by enterocytes and they are conveyed into plasma coupled to chylomicrons [104], aggravating inflammation. In this way, dietary fats can be associated with an increase in the absorption of LPS which is related to changes in the gut microbiota [105].

Inflammation is usually perceived as the connector among different comorbidities [106]. Another recent theory is that hypoxia could be a new potential risk factor for the chronic inflammation in obesity. The hypoxia is able to induce inflammation in adipose tissue by induction of gene expression in adipocytes and macrophages [107]. It is believed that local inflammation produced by hypoxia may serve as a physiological signal for angiogenesis, and remodeling of extracellular matrix in adipose tissue, although, when inflammation is out of control, it will promote insulin resistance locally and systemically [108]. During obesity, the enlargement of the vascular network is not sufficient to supply enough oxygen to all adipocytes and local hypoxia occurs [107], and this hypoxia could be a key trigger of adipose tissue dysfunction. In obesity, hypoxia appears in clusters of adipocytes that become distant from the vasculature as adipose tissue expands [109]. It has been established in mice with the deletion (VEGF (AdΔ)) that adipose vascular density is reduced and there is adipose hypoxia, while transgenic mice (VEGF (AdTg)) have increased adipose vasculature and reduced hypoxia [98], indicating the important role played by VEGF in this phenomenon.

Obesity increases the risk for T2D through induction of insulin resistance, and the link has been established with many factors such as inflammation, mitochondrial dysfunction, hyperinsulinemia, and lipotoxicity, but there is not a consensus about the mechanism of insulin resistance [110]. Inflammation could be seen as the best candidate; however, inflammation is not a good target in the treatment of insulin resistance as it has been corroborated in some clinical trials [111].

But as it can be noticed, none of the exposed theories can explain by themselves the complications in the metabolic disorders, and for that reason none of them has led to development of effective drugs and therapies. All the information must be taken into account together, trying to understand that each process interferes in the final status. A better understanding of the role of each of the parts of this orchestra will give us the capacity to understand the relationships and therefore the way and the level to act to obtain the best response.

## Abbreviations

T2D: Type 2 diabetes mellitus
IR: Insulin resistance
VAT: Visceral adipose tissue
SAT: Subcutaneous adipose tissue
FFA: Free fatty acids
PPAR*γ*: Peroxisome proliferator-activated receptor-*γ*
FASN: Fatty acid synthase
SCD: Stearoyl-CoA desaturase-1
BrCa1: Breast cancer 1
ZAG: Zinc-*α*2 glycoprotein
FABP4: Fatty acid-binding protein 4
SFRPs: Secreted frizzled-related proteins
SARPs: Secreted apoptosis-related proteins
THRSP or S14: Thyroid hormone responsive Spot 14
hO-MSC: Adipose tissue-derived multipotent stem cell population
BCL2: B-cell lymphoma
TWEAK: TNF-like weak inducer of apoptosis
TRAIL: TNF- (tumor necrosis factor-) related apoptosis-inducing ligand
VEGF: Vascular endothelial growth factor.
